# Correlation between oxygenation function and laboratory indicators in COVID-19 patients based on non-enhanced chest CT images and construction of an artificial intelligence prediction model

**DOI:** 10.3389/fmicb.2024.1495432

**Published:** 2024-11-06

**Authors:** Weiheng Kong, Yujia Liu, Wang Li, Keyi Yang, Lixin Yu, Guangyu Jiao

**Affiliations:** ^1^Department of Pulmonary and Critical Care Medicine, Shengjing Hospital of China Medical University, Shenyang, China; ^2^College of Traditional Chinese Medicine, Liaoning University of Traditional Chinese Medicine, Shenyang, China; ^3^Department of Radiology, Shengjing Hospital of China Medical University, Shenyang, China

**Keywords:** SARS-CoV-2, COVID-19, artificial intelligence, machine learning, chest CT radiomic features, PaO_2_/FiO_2_, laboratory indicators

## Abstract

**Objective:**

By extracting early chest CT radiomic features of COVID-19 patients, we explored their correlation with laboratory indicators and oxygenation index (PaO_2_/FiO_2_), thereby developed an Artificial Intelligence (AI) model based on radiomic features to predict the deterioration of oxygenation function in COVID-19 patients.

**Methods:**

This retrospective study included 384 patients with COVID-19, whose baseline information, laboratory indicators, oxygenation-related parameters, and non-enhanced chest CT images were collected. Utilizing the PaO_2_/FiO_2_ stratification proposed by the Berlin criteria, patients were divided into 4 groups, and differences in laboratory indicators among these groups were compared. Radiomic features were extracted, and their correlations with laboratory indicators and the PaO_2_/FiO_2_ were analyzed, respectively. Finally, an AI model was developed using the PaO_2_/FiO_2_ threshold of less than 200 mmHg as the label, and the model’s performance was assessed using the area under the receiver operating characteristic curve (AUC), sensitivity and specificity. Group datas comparison was analyzed using SPSS software, and radiomic features were extracted using Python-based Pyradiomics.

**Results:**

There were no statistically significant differences in baseline characteristics among the groups. Radiomic features showed differences in all 4 groups, while the differences in laboratory indicators were inconsistent, with some PaO_2_/FiO_2_ groups showed differences and others not. Regardless of whether laboratory indicators demonstrated differences across different PaO_2_/FiO_2_ groups, they could all be captured by radiomic features. Consequently, we chose radiomic features as variables to establish an AI model based on chest CT radiomic features. On the training set, the model achieved an AUC of 0.8137 (95% CI [0.7631–0.8612]), accuracy of 0.7249, sensitivity of 0.6626 and specificity of 0.8208. On the validation set, the model achieved an AUC of 0.8273 (95% CI [0.7475–0.9005]), accuracy of 0.7739, sensitivity of 0.7429 and specificity of 0.8222.

**Conclusion:**

This study found that the early chest CT radiomic features of COVID-19 patients are strongly associated not only with early laboratory indicators but also with the lowest PaO_2_/FiO_2_. Consequently, we developed an AI model based on CT radiomic features to predict deterioration in oxygenation function, which can provide a reliable basis for further clinical management and treatment.

## 1 Introduction

Coronavirus Disease 2019 (COVID-19) is caused by Severe Acute Respiratory Syndrome Coronavirus 2 (SARS-CoV-2) and has become a global pandemic threatening worldwide health (Sudre et al., 2021). SARS-CoV-2 infection can affect multiple organs and presents a variety of clinical symptoms (Wang et al., 2023). In the pathogenesis of COVID-19, a key factor is the dysregulation of immune inflammation (Xu et al., 2020). SARS-CoV-2 primarily enters respiratory epithelial cells by binding to angiotensin-converting enzyme 2 (ACE-2), triggering an immune inflammatory responses that results in varying degrees of damage to the alveolar epithelium, formation of hyaline membranes, and lung consolidation (Camporota et al., 2022; Caso et al., 2020; Qin et al., 2023). Therefore, the clinical symptoms of patients infected with COVID-19 exhibit significant heterogeneity; some patients are asymptomatic or exhibit only mild upper respiratory symptoms, while others may develop respiratory distress, potentially progressing to Acute Respiratory Distress Syndrome (ARDS) (Poston et al., 2020). The lungs are the organs most affected early and severely by COVID-19, and the rapid deterioration in respiratory function due to lung damage is a major cause of the high mortality rate in COVID-19 patients (Torres-Castro et al., 2021; Huang et al., 2020).

Clinically, the PaO_2_/FiO_2_ is used to represent oxygenation function and serves as a reliable predictor of acute lung injury (Matsubara et al., 2024). Since oxygenation dysfunction is an independent risk factor for progression to severe/critical COVID-19, deterioration in the PaO_2_/FiO_2_ provides an important basis for early clinical identification of worsening conditions in COVID-19 patients (Zhang et al., 2021). However, some critically ill patients may have mild clinical manifestations early in the disease, which do not correspond to the degree of oxygenation dysfunction due to severe lung damage (Tobin et al., 2020). Several laboratory indicators, such as lymphocytes, neutrophils, and pro-inflammatory cytokines, have been studied for predicting disease worsening and severe outcomes in COVID-19 patients (Del Valle et al., 2020; Zhao et al., 2020). Although these indicators reflect the immune-inflammatory status after SARS-CoV-2 infection, they are not directly indicative of oxygenation function and the extent of lung damage. Research by Fatima N et al. suggested a good correlation between early chest CT images and the PaO_2_/FiO_2_ in COVID-19 patients, indicating that chest CT can effectively assess the extent of lung damage and has potential for predicting severe cases of COVID-19 (Fatima et al., 2023; Liu F. et al., 2020).

Currently, semi-quantitative chest CT scoring systems have been developed to predict the severity and clinical outcomes of COVID-19 patients. However, these systems require radiologists to visually assess all chest CT images, which introduces considerable human error and prevents precise assessment (Wasilewski et al., 2020). Additionally, manual annotation of all infected areas for training leads to a substantial workload, making routine application challenging (Arian et al., 2023). To improve the sensitivity of COVID-19 assessment, AI-assisted quantitative analysis of chest CT is emerging as a new trend (Shaikh et al., 2021). Limited existing AI studies have extracted features such as lung lesion volume, inflammation area, and lesion density from chest CT images, with sample sizes generally around 100 cases, which limits comprehensive assessment of lung damage (Zhang et al., 2020; Pu et al., 2021; Pang et al., 2021). There is a pressing need to extract more lung features from larger samples to develop AI models that meet clinical needs for predicting severe lung damage in COVID-19 patients. Currently, researches based on AI primarily focus on employing AI techniques to analyze the different imaging findings presented in chest CT images of COVID-19 patients in order to predict disease severity and prognosis (Arian et al., 2023; Cai et al., 2020). There is a lack of comparative studies regarding oxygenation function and chest CT images using AI.

Therefore, this study will analyze the early chest CT radiomic features of COVID-19 patients using the PaO_2_/FiO_2_ as a stratification standard, exploring the correlation between early laboratory indicators, early chest CT radiomic features, and the PaO_2_/FiO_2_. We aim to establish an AI model to predict the extent of lung injury and deterioration in oxygenation function, providing a reliable basis for the early clinical management and treatment of COVID-19 patients.

## 2 Methods

### 2.1 Study subjects and clinical data

This retrospective study included patients admitted to our hospital from January 1, 2023, to June 1, 2024, with a diagnosis of novel coronavirus infection.

Inclusion criteria:

1. Diagnosed with novel coronavirus infection upon admission (National Health Commission, 2023).
2. Underwent CT examination on the day of admission and multiple blood gas analyses during the hospital stay.
3. Aged ≥18 years.

Cases that may interfere with this study or where obtaining imaging data is challenging will be excluded, including:

1. Patients requiring mechanical ventilation.
2. Pregnant patients or those with end-stage cancer.
3. Patients with concurrent pulmonary diseases such as pneumothorax, pulmonary edema, or mediastinal emphysema.
4. Patients with severe cardiac or renal dysfunction.
5. Patients with incomplete clinical data (Patients with incomplete laboratory indicators relevant to this study).
6. Patients with failed image acquisition (Cases with poor image quality or missing key frames during the CT imaging process).

**Figure 1:**
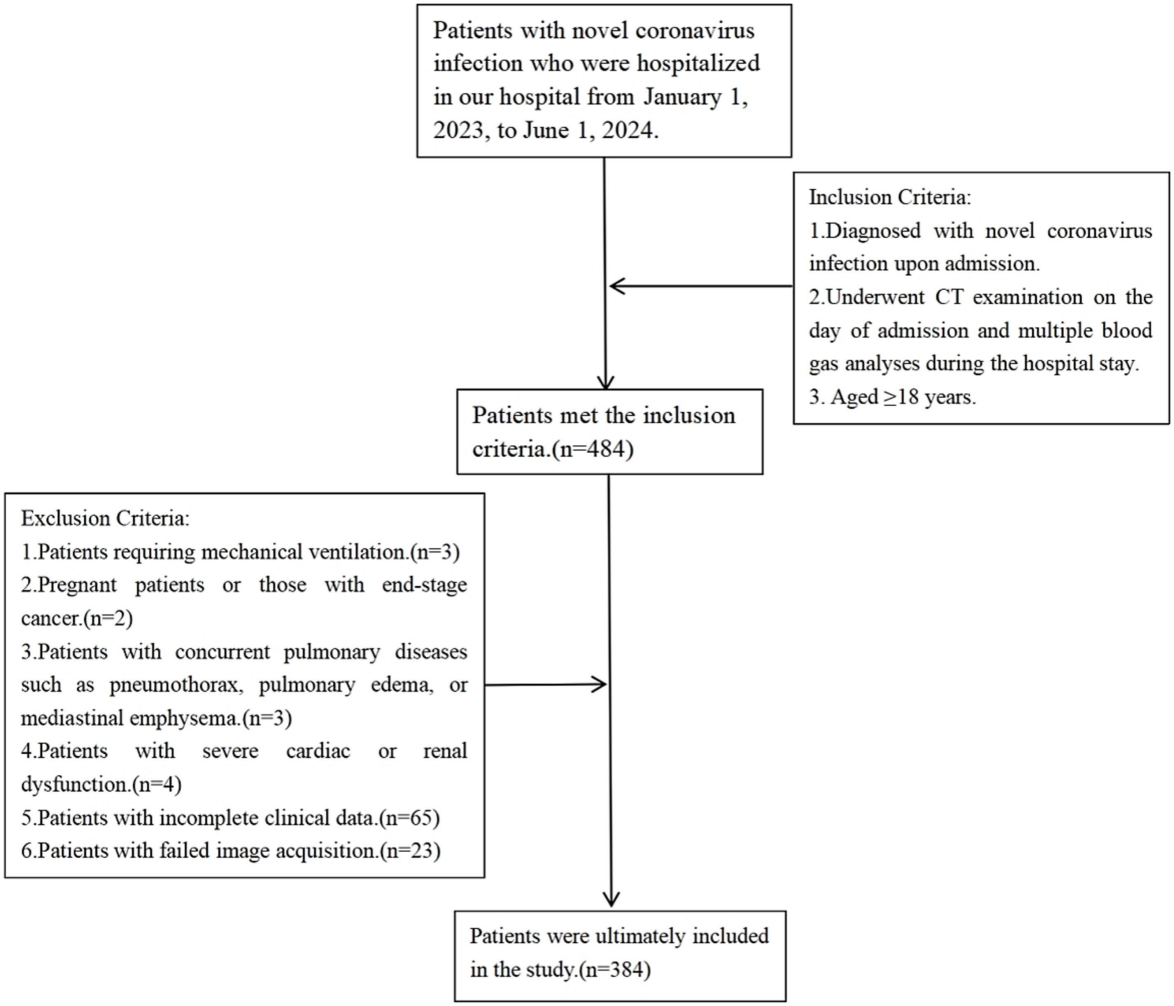
Flowchart for inclusion and exclusion of patients.

A total of 384 patients were ultimately included in the study. The flowchart for the inclusion and exclusion of patients is shown in Figure 1.

Relevant clinical and laboratory data from the included patients will be collected, including: Baseline Characteristics: Age, sex, BMI, smoking history, and comorbidities. Blood Gas Analysis and PaO_2_/FiO_2_: Blood gas analysis results (partial pressure of oxygen, PaO_2_), oxygen concentration (FiO_2_), and calculation of the PaO_2_/FiO_2_, with the lowest PaO_2_/FiO_2_ during hospitalization recorded. Laboratory Indicators on Admission Day: White blood cell count, neutrophil count and percentage, lymphocyte count and percentage, platelet count, C-reactive protein (CRP), D-dimer, lactate dehydrogenase (LDH), interleukin-6 (IL-6), ferritin, liver function indicators (AST, ALT), and cardiac indicators (B-type natriuretic peptide (BNP), troponin). Composite indicators such as the neutrophil-to-lymphocyte ratio (NLR), platelet-to-lymphocyte ratio (PLR), and systemic immune-inflammation index (SII) = platelet count × NLR were also calculated.

Quality control measures for laboratory indicators include: all operators complied with operational procedures, with no human-induced errors. The experimental instruments were all within their calibration periods. Reagents, quality control materials, and calibration standards for each indicator were all within their expiration dates and were properly stored. The laboratory environment’s temperature and humidity were maintained within acceptable ranges. During the experiments, all indicators passed quality control, with no random or systematic errors observed.

### 2.2 CT imaging protocol

Chest non-enhanced CT imaging was performed on the day of admission. All scans were conducted in the supine position with the patient in the inspiratory phase. The CT scans were performed using a Philips Brilliance ICT 256-slice spiral CT scanner, with the scanning range extending from the lung apex to the level of the costophrenic angle. Scanning parameters commonly used in our center included: tube voltage of 120 kV, tube current adjusted automatically, matrix of 512 × 512, pitch of 1, conventional image thickness of 3.0 mm, and thin-slice images with 1.0 mm intervals for 3D reconstruction.

### 2.3 Lung segmentation and features extraction

**Figure 2:**
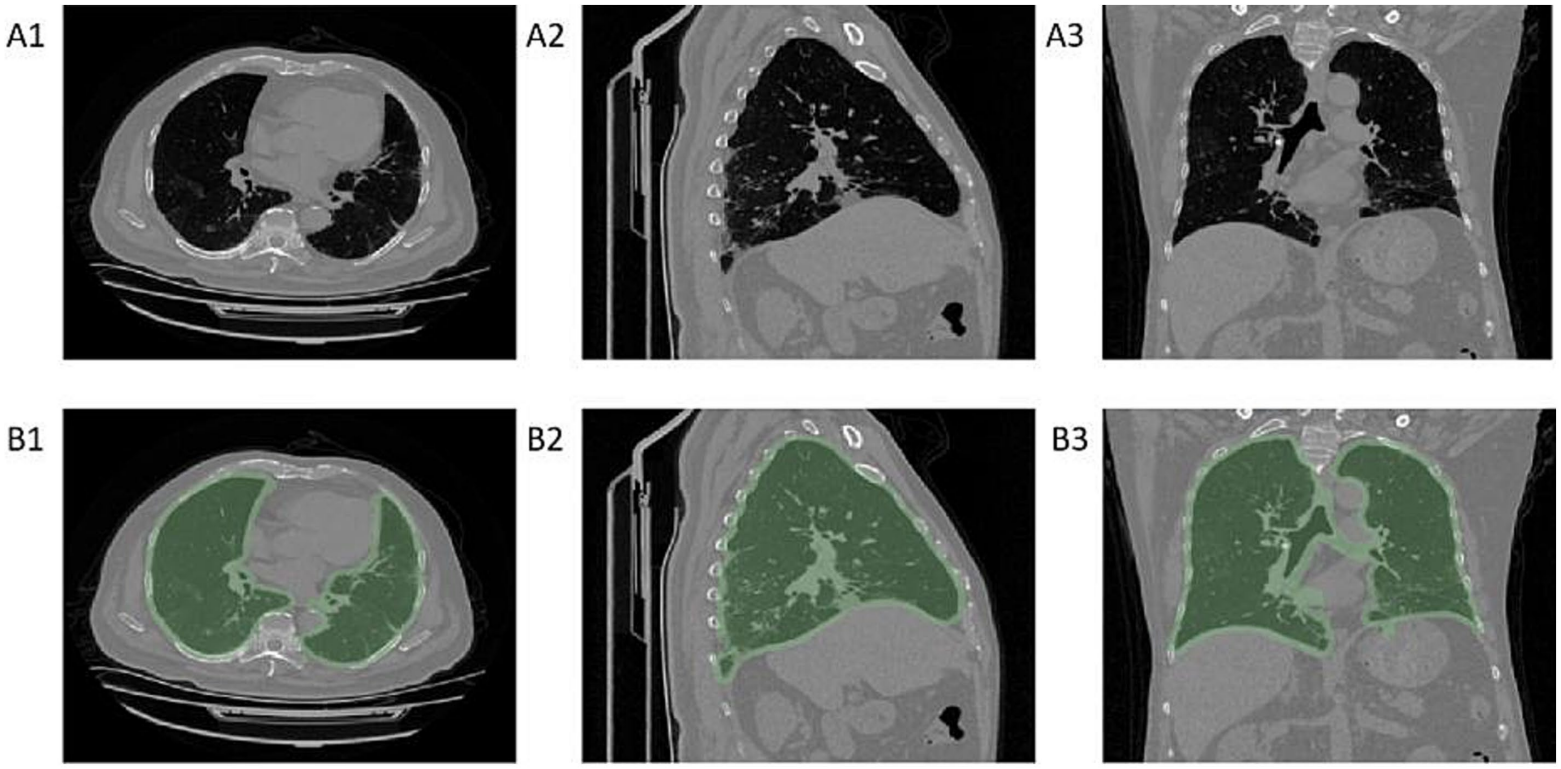
Diagram of lung segmentation. Panels **A1–A3** show chest CT cross-sectional images, while panels **B1–B3** display the regions of interest (ROI) for the lungs identified by the machine learning model on chest CT. **A1–B1** are axial CT images, **A2–B2** are sagittal CT images, and **A3–B3** are coronal CT images.

To reduce the interference of extrathoracic factors on the model, we developed a machine learning segmentation algorithm for lung segmentation. The image matrix values were first converted to attenuation values for CT images, and pixels with attenuation values less than −700 were used as a mask. After image erosion, only the largest connected domain was retained, and the mask was then expanded again to determine it as the region of interest (ROI) for the lungs, as shown in Figure 2.

Radiomic features were extracted using Python-based Pyradiomics. Prior to feature extraction, the segmented images were preprocessed to minimize the impact of contrast and brightness variations on the radiomic features. A total of 944 radiomic features were generated for each patient, based on first-order (*n* = 18), shape (*n* = 14), texture (*n* = 75), Gaussian Laplacian filters (*n* = 93), and wavelet filters (*n* = 744).

### 2.4 Machine learning

In clinical practice, patients with PaO_2_/FiO_2_ less than 200 mmHg are considered to have moderate to severe ARDS and usually require mechanical ventilation (Ranieri et al., 2012; Qadir et al., 2024). Therefore, we used PaO_2_/FiO_2_ 200 mmHg as the grouping criterion, dividing patients into a mechanical ventilation group (PaO_2_/FiO_2_ ≤ 200 mmHg) and a non-mechanical ventilation group (PaO_2_/FiO_2_ > 200 mmHg). The machine learning models for this study, developed using the Python sklearn library, employed various machine learning methods to predict the aforementioned labels. Model performance was evaluated using the area under the receiver operating characteristic curve (AUC), sensitivity and specificity. Internal validation was used to assess the machine learning models. During model development, the entire dataset was randomly divided into training and validation sets, and five-fold cross-validation was used for model validation.

In this study, we employed the Linear Discriminant Analysis (LDA) algorithm, a form of supervised learning, for dimensionality reduction and essential feature extraction. We extracted over 900 radiomic features for each patient in the study. By utilizing this algorithm, we aimed to reduce the number of features in the input data, enabling the representation of the output affecting labels with a minimal set of features. The fundamental concept is to project the training sample set onto a single line in such a way that the projection points of samples from the same class are as close together as possible, while the centers of the projection points from different classes are as far apart as possible (Xu et al., 2022).

### 2.5 Statistical analysis

Statistical analysis was performed using IBM SPSS 27.0 software. All data were tested for normality; normally distributed quantitative data were described as mean ± standard deviation, while non-normally distributed quantitative data were described as median (interquartile range). The Kruskal-Wallis test was used for comparing multiple groups, and the Mann–Whitney U test was used for multiple comparisons among groups. Categorical data were described using frequencies (percentages) and compared using the chi-square (*χ*2) test. A *p*-value of <0.05 was considered statistically significant. Statistical plots were generated using Python-based matplotlib.

## 3 Results

### 3.1 Basic characteristics

A total of 384 patients were included in this study. Table 1 presents the clinical characteristics of the included patients. The median age of all patients was 71.00 (62.25, 78.00) years, and the median BMI was 24.29 (22.19, 26.88). Among the patients, 227 (59.1%) were male and 157 (40.9%) were female. A total of 105 patients (27.3%) had a history of smoking. The most common comorbidities among the included patients were hypertension, diabetes, and cardiovascular diseases. Based on the Berlin definition guidelines for ARDS (Ranieri et al., 2012), patients were divided into four groups according to their PaO_2_/FiO_2_: PaO_2_/FiO_2_ > 300 mmHg, PaO_2_/FiO_2_ 200-300 mmHg, PaO_2_/FiO_2_ 100-200 mmHg, and PaO_2_/FiO_2_ ≤ 100 mmHg.

**Table 1:** The clinical characteristics of the included patients.

	Characteristics	Statistical value
Cases number		384
Sex		
	Male	227 (59.1%)
	Female	157 (40.9%)
Age		71.00 (62.25, 78.00)
BMI		24.29 (22.19, 26.88)
Smoking history		
	Yes	105 (27.3%)
	No	279 (72.7%)
Comorbidity		
	Hypertension	184 (47.9%)
	Diabetes	97 (25.3%)
	Cardiovascular disease	76 (19.8%)
	COPD	5 (1.3%)
	Chronic kidney disease	23 (6.0%)
PaO_2_/FiO_2_ Grouping		
	>300 mmHg	106 (27.6%)
	200-300 mmHg	127 (33.1%)
	100-200 mmHg	119 (31.0%)
	≤100 mmHg	32 (8.3%)

There were no statistically significant differences in sex, age, BMI, smoking history, or comorbidities among the four patient groups (*p* > 0.05). See Table 2.

**Table 2:** Comparison of baseline characteristics among four groups of patients.

Characteristics	>300 mmHg *n* = 106	200-300 mmHg *n* = 127	100-200 mmHg *n* = 119	≤100 mmH *n* = 32	Statistical value	*p* value
Sex					*χ*2 = 3.927	0.269
Male	61 (59.1%)	68 (53.5%)	78 (65.5%)	20 (62.5%)		
Female	45 (42.5%)	59 (46.1%)	41 (34.5%)	12 (37.5%)		
Age	68.50 (60.00, 74.25)	71.00 (64.00, 78.00)	71.00 (63.00, 79.00)	72.00 (67.25, 76.75)	*H* = 7.676	0.053
BMI	23.75 (22.15, 26.83)	24.77 (21.87, 27.17)	24.22 (22.22, 26.89)	25.23 (22.71, 26.73)	*H* = 1.246	0.742
Smoking history					*χ*2 = 4.201	0.241
Yes	25 (23.6%)	30 (23.6%)	39 (32.8%)	11 (34.4%)		
No	84 (76.4%)	97 (76.4%)	80 (67.2%)	21 (65.6%)		
Comorbidities						
Hypertension	46 (43.4%)	58 (45.7%)	59 (49.6%)	21 (65.6%)	*χ*2 = 5.278	0.153
Diabetes	20 (18.9%)	32 (25.2%)	32 (26.9%)	13 (40.6%)	*χ*2 = 6.463	0.091
Cardiovascular disease	13 (12.3%)	27 (21.3%)	26 (21.8%)	10 (31.3%)	*χ*2 = 6.920	0.074
COPD	1 (0.9%)	2 (1.6%)	2 (1.7%)	0 (0.0%)	*χ*2 = 0.735	0.865
Chronic kidney disease	6 (5.7%)	7 (5.5%)	7 (5.9%)	3 (9.4%)	*χ*2 = 0.727	0.867

**Figure 3:**
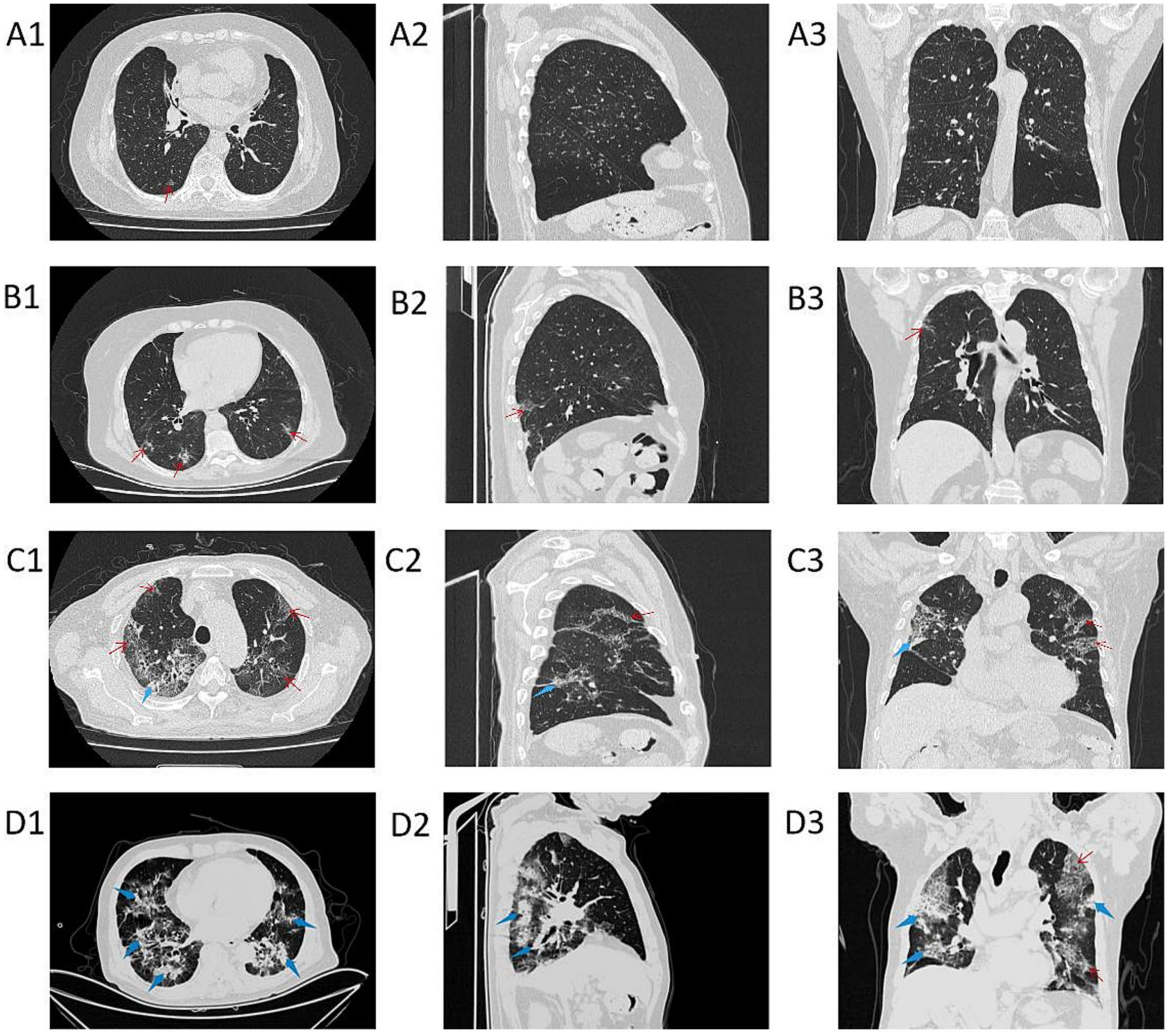
Chest CT images of patients in different PaO_2_/FiO_2_ groups. **A1–A3** are chest CT images of patients with PaO_2_/FiO_2_ > 300 mmHg; **B1–B3** are chest CT images of patients with PaO_2_/FiO_2_ 200-300 mmHg; **C1–C3** are chest CT images of patients with PaO_2_/FiO_2_ 100-200 mmHg; **D1–D3** are chest CT images of patients with PaO_2_/FiO_2_ ≤ 100 mmHg. **A1–D1** show axial CT; **A2–D2** show sagittal CT; **A3–D3** show coronal CT. The red thin arrows indicate ground-glass opacities (GGOs) and interlobular septal thickening; the blue thick arrows indicate consolidation.

As the PaO_2_/FiO_2_ decreases, the range and density of lung lesions in the chest CT images increase. In the PaO_2_/FiO_2_ > 300 mmHg group, patients exhibit a few scattered exudative lesions in the lungs (see Figures 3A1–A3). In the PaO_2_/FiO_2_ 200-300 mmHg group, patients show fewer lung lesions, primarily ground-glass opacities (GGOs) with limited extent (see Figures 3B1–B3). In the PaO_2_/FiO_2_ 100-200 mmHg group, patients have a larger number of lung lesions, including GGOs and some consolidation, with a more extensive distribution (see Figures 3C1–C3). In the PaO_2_/FiO_2_ ≤ 100 mmHg group, patients present with dense lung lesions, including diffuse consolidation, with widespread distribution throughout the lungs (see Figures 3D1–D3).

### 3.2 Analysis of differences in radiomic features across different PaO_2_/FiO_2_ groups

**Figure 4:**
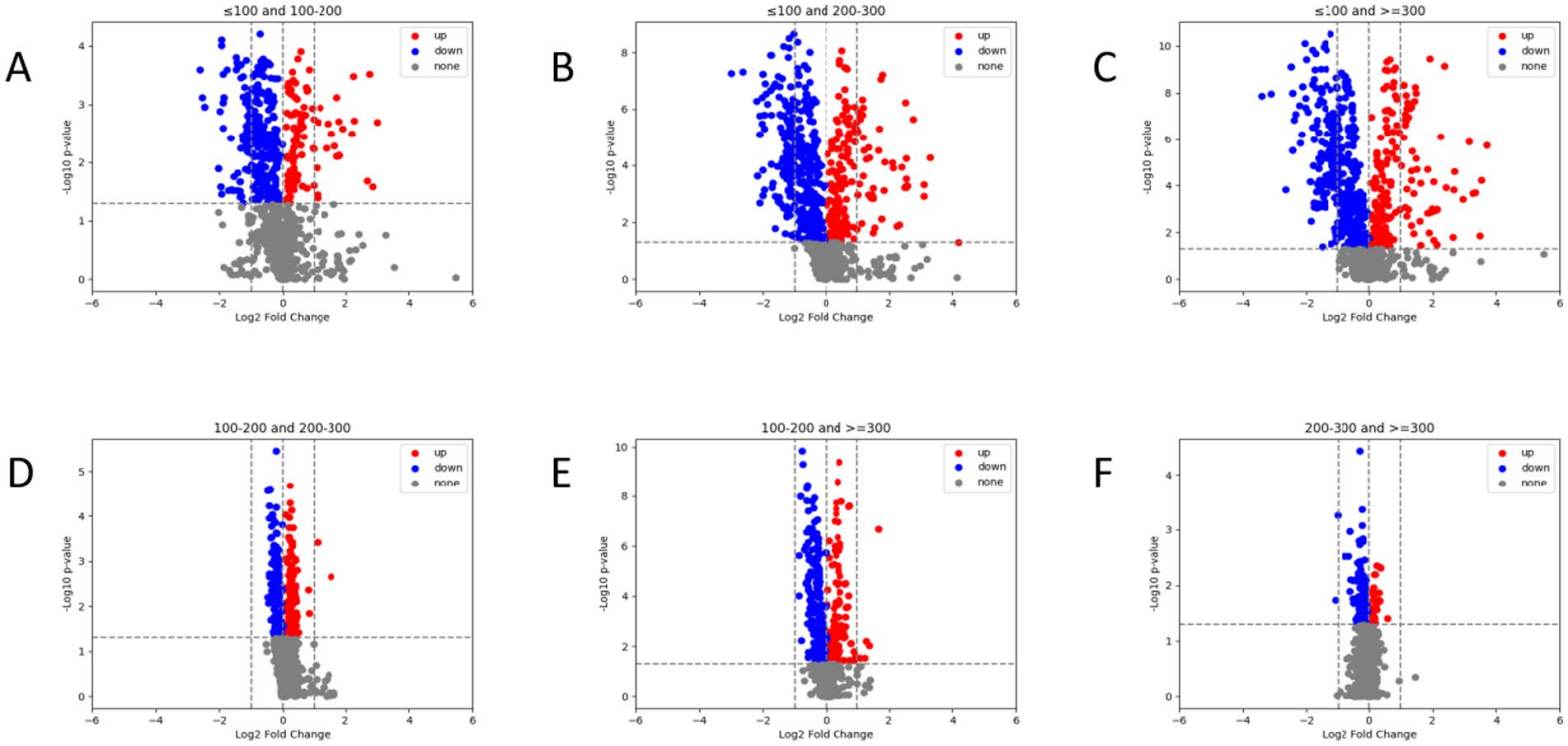
Comparison of radiomic features between different PaO_2_/FiO_2_ groups via volcano plot. There are significant differences in radiomic feature expressions between different PaO_2_/FiO_2_ groups **(A–F)**. Patients in the PaO_2_/FiO_2_ ≤ 100 mmHg group show the most pronounced differences in radiomic features compared to the other three groups **(A–C)**. The differences in radiomic features between the PaO_2_/FiO_2_ 100-200 mmHg group and the PaO_2_/FiO_2_ 200-300 mmHg group, as well as the PaO_2_/FiO_2_ ≥ 300 mmHg group, are notable **(D,E)**. There are differences in radiomic features between the PaO_2_/FiO_2_ 200-300 mmHg group and the PaO_2_/FiO_2_ ≥ 300 mmHg group, but the differences are small **(F)**. In the figure, blue points and red points represent significant differences, while gray points indicate no difference. A higher number of points indicates a greater degree of difference.

As shown in Figure 4, we compared the differences in radiomic feature expressions among different groups. There are significant differences in the radiomic features among patients in different PaO_2_/FiO_2_ groups (*p* < 0.05). Specifically, patients in the PaO_2_/FiO_2_ ≤ 100 mmHg group show the most pronounced differences in radiomic features compared to the other three groups (Figures 4A–C). As the PaO_2_/FiO_2_ increases, the differences in radiomic features gradually decrease (Figures 4D–F).

### 3.3 Analysis of laboratory indicators across different PaO_2_/FiO_2_ groups

Comparing laboratory indicators across different PaO_2_/FiO_2_ groups, we observed statistical differences in immune-inflammatory indicators, coagulation indicators, and cardiac-related indicators among the four groups (*p* < 0.05). However, no statistical differences were found in platelet counts and liver-related indicators (AST, ALT) (*p* > 0.05) (see Table 3).

**Table 3:** Differential analysis of laboratory indicators among four groups of patients.

Laboratory indicators	>300 mmHg *n* = 106	200-300 mmHg *n* = 127	100-200 mmHg *n* = 119	≤100 mmHg *n* = 32	Statistical value	*p* value
Immune-inflammatory indicators						
White blood cell count (10^9^/L)	6.52 (5.14, 8.69)	7.00 (4.83, 9.12)	7.20 (5.48, 9.98)	8.44 (6.45, 12.51)	*H* = 13.649	**0.003**
Neutrophil percentage (%)	69.90 (62.85, 80.60)	76.50 (68.40, 85.30)	80.20 (71.70, 87.60)	86.70 (79.93, 91.13)	*H* = 45.212	**<0.001**
Neutrophil count (10^9^/L)	4.60 (3.40, 6.30)	5.10 (3.20, 7.00)	5.90 (4.10, 8.10)	7.55 (5.10, 11.23)	*H* = 27.300	**<0.001**
Lymphocyte percentage (%)	19.30 (11.03, 26.53)	14.80 (8.50, 20.30)	10.30 (5.90, 16.90)	5.75 (3.48, 12.15)	*H* = 55.444	**<0.001**
Lymphocyte count (10^9^/L)	1.20 (0.70, 1.53)	0.90 (0.60, 1.40)	0.80 (0.50, 1.10)	0.55 (0.40, 0.90)	*H* = 28.781	**<0.001**
CRP (mg/L)	15.55 (5.46, 48.01)	25.40 (9.60, 62.00)	38.90 (9.70, 87.30)	71.99 (27.21, 100.87)	*H* = 23.440	**<0.001**
LDH (U/L)	270.00 (21.75, 301.00)	297.00 (242.00, 309.00)	301.00 (280.00, 343.00)	341.00 (287.25, 430.25)	*H* = 37.108	**<0.001**
IL-6 (pg/mL)	7.12 (2.39, 16.27)	11.55 (3.17, 20.35)	12.90 (3.95, 27.02)	16.85 (7.65, 30.82)	*H* = 16.268	**<0.001**
Ferritin (ng/mL)	316.50 (226.48, 514.48)	387.00 (241.10, 537.50)	486.40 (267.00, 573.50)	537.50 (367.95, 705.13)	*H* = 18.150	**<0.001**
NLR	3.6667 (2.4152, 7.5000)	4.8889 (3.2500, 10.0000)	7.7500 (4.4444, 15.5000)	14.0417 (6.4560, 24.5000)	*H* = 53.106	**<0.001**
PLR	184.7802 (117.3438, 291.2500)	202.5000 (146.1905, 350.0000)	283.3333 (173.3333, 410.0000)	294.1667 (204.5000, 512.5000)	*H* = 27.752	**<0.001**
SII	763.0833 (464.8472, 1605.5000)	1147.0000 (540.5714, 2141.6667)	1515.5556 (854.0000, 2908.8889)	2527.0000 (1205.5130, 6080.9583)	*H* = 41.347	**<0.001**
Coagulation indicators						
platelet count (10^9^/L)	200.00 (149.75, 258.00)	199.00 (142.00, 261.00)	207.00 (148.00, 287.00)	179.00 (141.00, 257.75)	*H* = 1.426	0.699
D-dime r (μg/L)	180.00 (96.75, 425.50)	215.00 (133.00, 499.00)	264.00 (160.00, 486.00)	599.00 (257.75, 2818.75)	*H* = 29.910	**<0.001**
Liver-related indicators						
AST (U/L)	21.50 (16.00, 31.75)	22.00 (15.00, 33.00)	24.00 (17.00, 35.00)	27.00 (22.00, 44.00)	*H* = 7.235	0.065
ALT (U/L)	23.00 (16.00, 37.50)	27.00 (18.00, 43.00)	28.00 (18.00, 42.00)	34.00 (18.50, 50.75)	*H* = 4.934	0.177
Cardiac-related indicators						
BNP (pg/mL)	50.15 (17.28, 82.03)	69.00 (16.30, 100.31)	80.30 (35.30, 122.00)	67.25 (33.93, 146.40)	*H* = 16.990	**<0.001**
Troponin (μg/L)	0.0056 (0.0038, 0.0088)	0.0072 (0.0041, 0.0113)	0.0089 (0.0058, 0.0160)	0.0120 (0.0077, 0.0345)	*H* = 36.531	**<0.001**

The values in bold indicate that the *p*-values are less than 0.05, meaning there are significant differences among the four groups of data with statistical significance.

**Figure 5:**
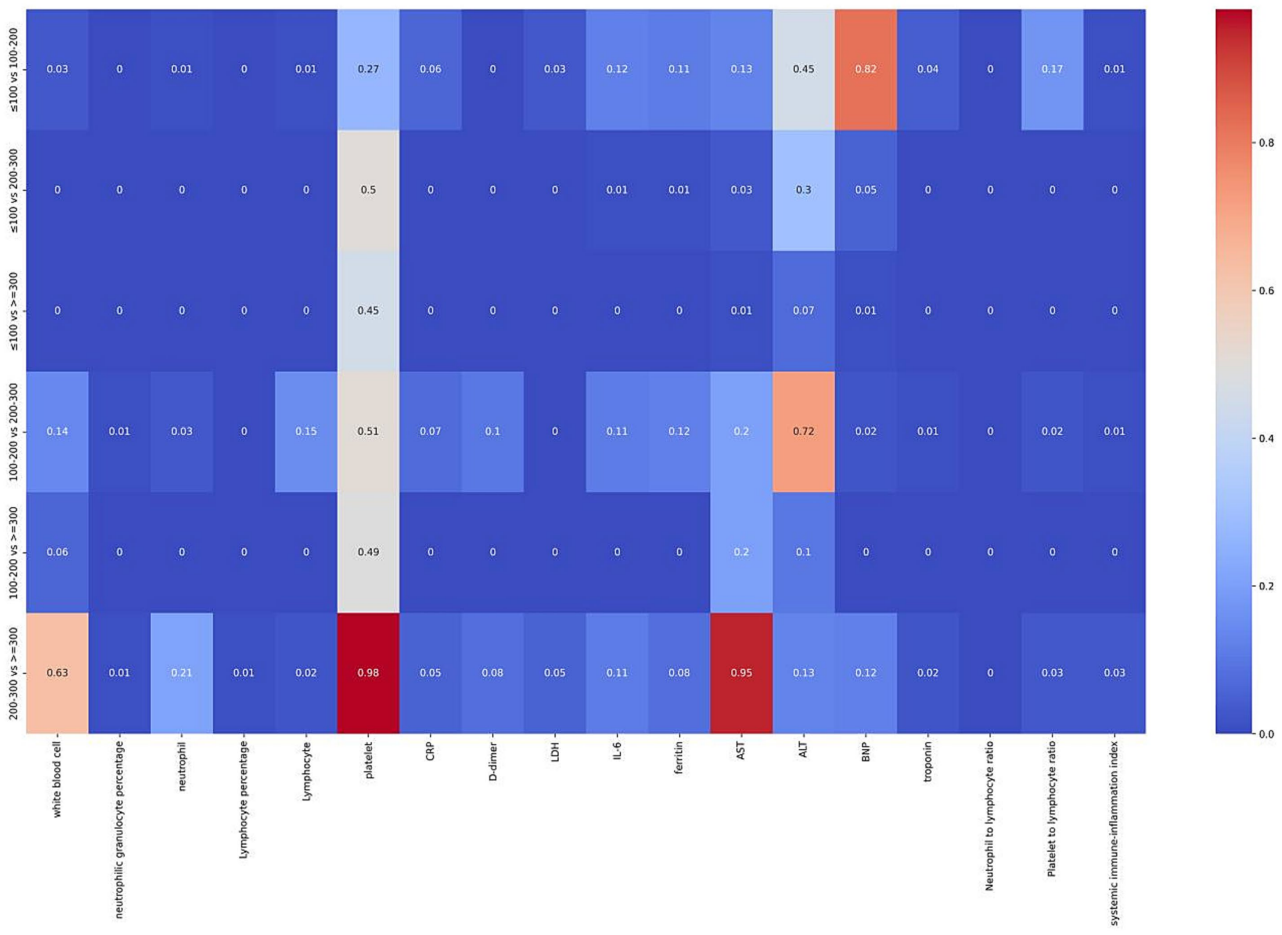
Heatmap of differences in laboratory indicators between pairs of PaO_2_/FiO_2_ groups. The x-axis represents laboratory indicators, and the y-axis represents pairwise comparisons between PaO_2_/FiO_2_ groups. Differences were analyzed using the Mann–Whitney *U* test. The bar in the figure indicates the *p*-value (0–1), with *p* < 0.05 indicating statistical significance and *p* > 0.05 indicating no statistical significance.

To clarify the specific differences between groups, we performed pairwise post-hoc comparisons (see Figure 5). We found statistically significant differences in neutrophil percentage, lymphocyte percentage, LDH, NLR, SII, and troponin across different PaO_2_/FiO_2_ groups (*p* < 0.05).

However, the differences in laboratory indicators such as white blood cells, neutrophils, lymphocytes, CRP, IL-6, ferritin, D-dimer, BNP, and PLR varied inconsistently among the PaO_2_/FiO_2_ groups. Specifically, differences in these indicators between the PaO_2_/FiO_2_ ≤ 100 mmHg group and the PaO_2_/FiO_2_ 200-300 mmHg group, as well as the PaO_2_/FiO_2_ > 300 mmHg group, were statistically significant (*p* < 0.05). Conversely, white blood cells, D-dimer, IL-6, and ferritin showed no significant differences between the PaO_2_/FiO_2_ 200-300 mmHg group and the PaO_2_/FiO_2_ > 300 mmHg group, or between the PaO_2_/FiO_2_ 200-300 mmHg group and the PaO_2_/FiO_2_ 100-200 mmHg group (*p* > 0.05). Therefore, the direct correlation between laboratory indicators and PaO_2_/FiO_2_ is not clear, and these indicators cannot accurately reflect the PaO_2_/FiO_2_ status.

**Figure 6:**
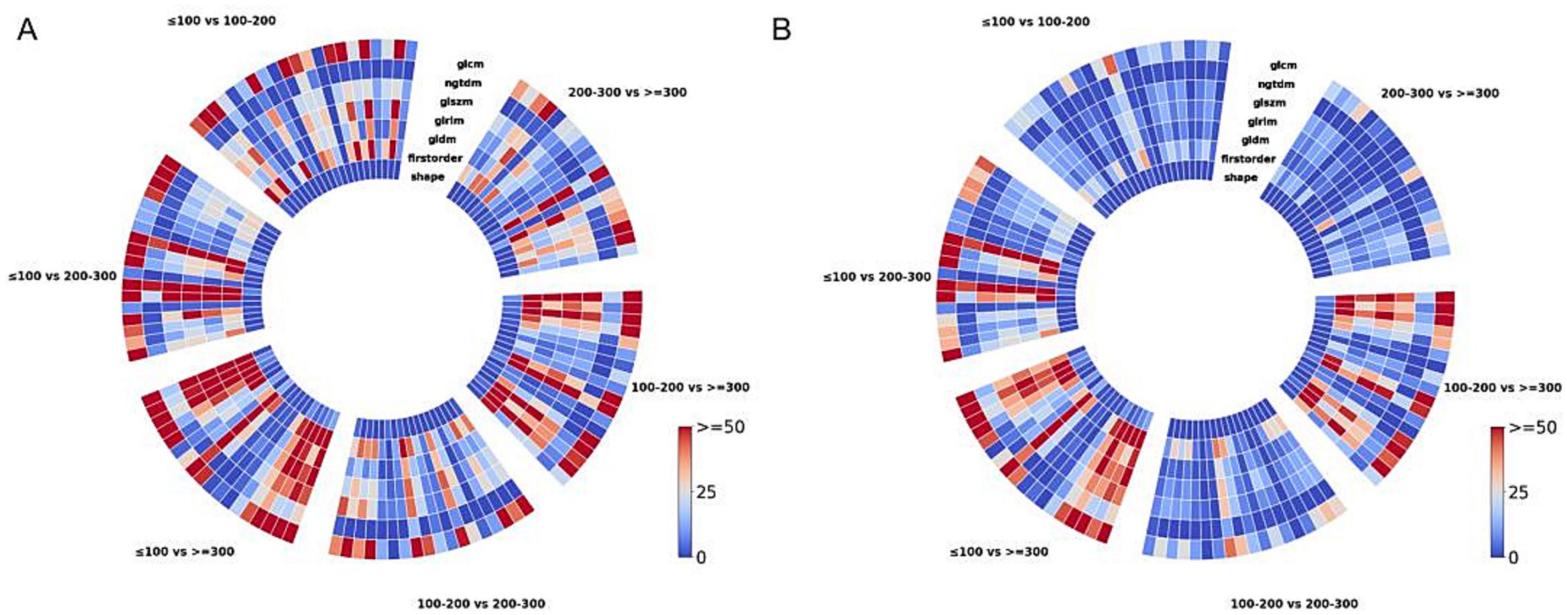
Texture feature map showing the correlation between laboratory indicators and radiomic features. In pairwise comparisons of different PaO_2_/FiO_2_ groups, different laboratory indicators are directly captured by the intensity of radiomic features **(A)**. Radiomic features that both capture laboratory indicators and show differences between different PaO_2_/FiO_2_ groups are illustrated in **(B)**.

As shown in Figure 6A, correlation analysis between the aforementioned laboratory indicators and radiomic features reveals that changes in laboratory indicators are directly reflected in the patients’ chest CT images and are sharply captured by radiomic features. Among these radiomic features that can capture laboratory indicators, the majority show significant differences between different PaO_2_/FiO_2_ groups, regardless of whether the laboratory indicators themselves differ between the PaO_2_/FiO_2_ groups, as illustrated in Figure 6B.

### 3.4 Chest CT radiomic features model

In different PaO_2_/FiO_2_ groups, there are statistically significant differences in radiomic features. Additionally, regardless of whether laboratory indicators have differences between PaO_2_/FiO_2_ groups, they can be captured by radiomic features. Therefore, we selected only radiomic features as variables and established a chest CT radiomic features AI model. To efficiently and accurately predict whether COVID-19 patients require mechanical ventilation due to decreased PaO_2_/FiO_2_, we combined patients with PaO_2_/FiO_2_ ≤ 100 mmHg and 100-200 mmHg into the mechanical ventilation group, and those with PaO_2_/FiO_2_ 200-300 mmHg and > 300 mmHg into the non-mechanical ventilation group.

**Figure 7:**
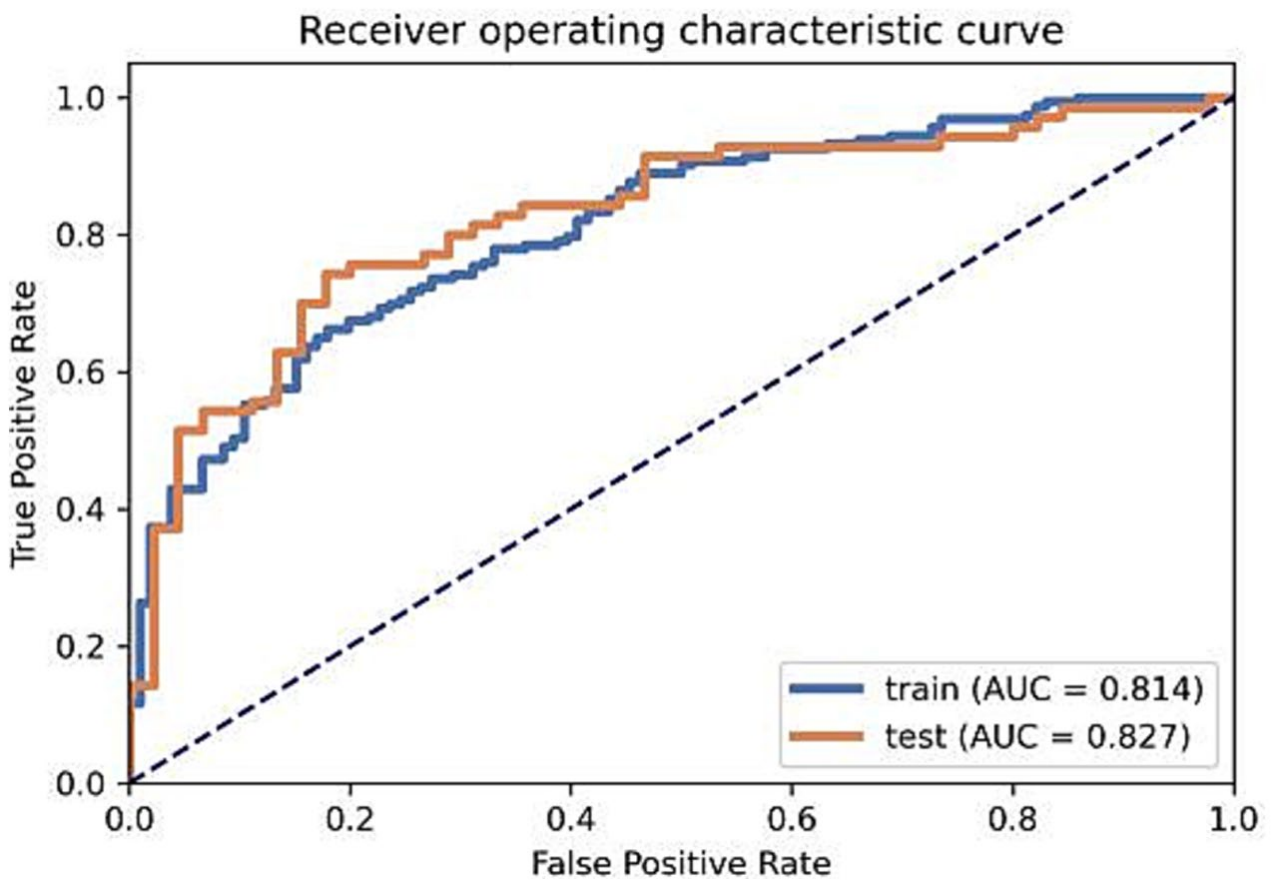
The ROC curves for the training and validation sets.

On the training set, the model’s AUC was 0.8137 (95% CI [0.7631–0.8612]), with an accuracy of 0.7249, sensitivity of 0.6626, and specificity of 0.8208. On the validation set, the model’s AUC was 0.8273 (95% CI [0.7475–0.9005]), with an accuracy of 0.7739, sensitivity of 0.7429, and specificity of 0.8222, as shown in Figure 7.

## 4 Discussion

This study is the first to use machine learning methods to segment lung ROIs and extract radiomic features from early chest CT images of over 380 COVID-19 patients. We utilized these radiomic features as intermediate variables to explore the direct and indirect correlations between laboratory indicators and PaO_2_/FiO_2_, thereby validating the assessment capability of CT—one of the most commonly used imaging modalities for COVID-19—of the overall physiological and pathological state represented by laboratory indicators. Finally, we developed an AI model based on early chest CT radiomic features of COVID-19 patients to predict whether mechanical ventilation would be required due to a decrease in PaO_2_/FiO_2_.

SARS-CoV-2 infection can trigger a robust immune response (Gallais et al., 2021). Early immune response in COVID-19 plays a protective role in viral clearance, whereas an excessive immune response can release an overabundance of pro-inflammatory cytokines and chemokines, leading to cytokine storms and systemic immune cascade reactions, which in turn alter laboratory immune-inflammatory indicators and coagulation indicators (Alzaabi et al., 2021; Chen, R. et al., 2020). Additionally, exacerbated and dysregulated immune responses can cause multi-organ damage, with the lungs being among the earliest and most severely affected organs (Chen N. et al., 2020). Researches by Liu and Fu et al. have demonstrated that laboratory indicators can be used to predict the overall deterioration and adverse outcomes in COVID-19 patients (Fu et al., 2020; Liu J. et al., 2020). However, to date, there have been no studies directly predicting the degree of lung injury and oxygenation function through the analysis of changes in laboratory indicators. To address this, we explored whether multiple laboratory indicators directly correlate with the PaO_2_/FiO_2_. Results showed that while there are overall differences in laboratory indicators across four different PaO_2_/FiO_2_ groups, the differences are inconsistent when comparing pairwise groups. For example, white blood cell count, CRP, IL-6, and ferritin were statistically significant between the PaO_2_/FiO_2_ ≤ 100 mmHg group and the PaO_2_/FiO_2_ 200-300 mmHg group, but there were no statistical differences between the PaO_2_/FiO_2_ 200-300 mmHg group and the PaO_2_/FiO_2_ > 300 mmHg group. These results suggest that laboratory indicators alone do not fully and accurately assess oxygenation function and the extent of lung damage in COVID-19 patients. Therefore, more appropriate assessment indicators are needed clinically.

We subsequently focused on early chest CT images to analyze their correlation with the PaO_2_/FiO_2_. We found significant differences in radiomic features among different PaO_2_/FiO_2_ groups, particularly in the patients with PaO_2_/FiO_2_ ≤ 100 mmHg, whose radiomic features showed a very significant difference compared to the other three groups. Thus, the radiomic features derived from non-contrast chest CT images may provide a valuable tool for predicting the PaO_2_/FiO_2_. Furthermore, we conducted a correlation analysis between radiomic features and laboratory indicators, revealing significant correlations between them. Notably, even laboratory indicators that were not directly related to the PaO_2_/FiO_2_ showed a strong association with the radiomic features, indicating that these features may also serve as accurate reflections of the body’s inflammatory response level.

This study is the first to extract radiomic features from early chest CT scans of over 380 COVID-19 patients, using the PaO_2_/FiO_2_ as the stratification criterion. We established an AI model based on early chest CT radiomic features, which achieve an accuracy of 0.8 in predicting stratification for the PaO_2_/FiO_2_ above and below 200 mmHg. Although AI-driven quantitative analysis of CT scans has shown promise in assessing clinical classifications, predicting disease progression, and evaluating sequelae in COVID-19 patients, the current research often relies on radiologists visually assessing and manually annotating CT images (Salahshour et al., 2021; Tanaka et al., 2023; Wasilewski et al., 2020). This heavy workload limits the ability to evaluate large samples, and reducing human error remains a significant challenge. Furthermore, studies utilizing AI technology for CT imaging primarily focus on identifying and analyzing specific features such as lesion volume, inflammatory area, or lesion density (Pang et al., 2021; Alilou et al., 2023; Chung et al., 2021). This narrow focus may lead to incomplete assessments and, similarly, suffers from issues related to high error margins and low accuracy.

This study departs from traditional visual assessment methods by disruptively applying computer programming languages to extract over 900 radiomic numerical features from CT images, including first-order, shape, texture, Gaussian Laplacian filters, and wavelet filters. Using machine learning for training and validation, we ultimately selected the feature parameter combinations most strongly correlated with the PaO_2_/FiO_2_ to construct a CT-AI model for lung assessment, achieving high accuracy and specificity in predicting oxygenation function. Clinically, patients with an PaO_2_/FiO_2_ below 200 mmHg generally require mechanical ventilation (Qadir et al., 2024). Santus P and Zhou W have confirmed that an PaO_2_/FiO_2_ < 200 mmHg at admission is independently associated with higher mortality, which can help clinicians identify high-risk patients early in their hospital stay (Santus et al., 2020; Zhou et al., 2021). Therefore, we selected PaO_2_/FiO_2_ 200 mmHg as the threshold value in clinical practice, dividing patients into two groups: the mechanical ventilation group (including the PaO_2_/FiO_2_ ≤ 100 mmHg group and the PaO_2_/FiO_2_ 100–200 mmHg group) and the non-mechanical ventilation group (including the PaO_2_/FiO_2_ 200–300 mmHg group and the PaO_2_/FiO_2_ > 300 mmHg group). The results indicate that this model can predict stratification tasks with an accuracy of 0.8 for determining whether the PaO_2_/FiO_2_ is above or below 200 mmHg. This capability can assist clinicians in automatically identifying high-risk patients through early admission CT scans, effectively guiding the monitoring of critically ill patients, the need for increased oxygen supplementation, and decisions regarding mechanical ventilation.

This study does have some limitations. First, it is a single-center study, lacking multi-center data to further validate these conclusions. Second, the study only explored the AI model’s ability to predict the lowest PaO_2_/FiO_2_ during hospitalization, lacking comprehensive monitoring throughout the patient’s disease course, the further model can be established for dynamic monitoring and the prediction of the long COVID-19 in the future. Third, we used only the PaO_2_/FiO_2_ as the primary parameter for assessing COVID-19 severity, without considering other complications that may arise during the disease course. Finally, given the high heterogeneity of COVID-19, future research will further explore their corresponding mechanism and the impact of genetic susceptibility on the PaO_2_/FiO_2_.

## 5 Conclusion

This study found that the early chest CT radiomic features of COVID-19 patients show a strong correlation with early laboratory indicators and the lowest PaO_2_/FiO_2_. Therefore, we established an AI model based on the early chest CT radiomic characteristics of COVID-19 patients, which can be used to predict the deterioration of oxygenation function in COVID-19 patients, providing a basis for selecting further clinical management and treatment measures.

## Data Availability

The original contributions presented in the study are included in the article/Supplementary material, further inquiries can be directed to the corresponding author.

## References

[ref1] Alilou S. Zangiabadian M. Pouramini A. Jaberinezhad M. Shobeiri P. Ghozy S.. (2023). Radiological findings as predictors of COVID-19 lung sequelae: a systematic review and Meta-analysis. Acad. Radiol. 30, 3076–3085. doi: 10.1016/j.acra.2023.06.002, PMID: 37491177 PMC10242153

[ref2] Alzaabi A. H. Ahmed L. A. Rabooy A. E. Zaabi A. A. Alkaabi M. AlMahmoud F.. (2021). Longitudinal changes in IgG levels among COVID-19 recovered patients: a prospective cohort study. PLoS One 16:e0251159. doi: 10.1371/journal.pone.0251159, PMID: 34115768 PMC8195379

[ref3] Arian A. Mehrabi Nejad M. M. Zoorpaikar M. Hasanzadeh N. Sotoudeh-Paima S. Kolahi S.. (2023). Accuracy of artificial intelligence CT quantification in predicting COVID-19 subjects' prognosis. PLoS One 18:e0294899. doi: 10.1371/journal.pone.0294899, PMID: 38064442 PMC10707659

[ref4] Cai W. Liu T. Xue X. Luo G. Wang X. Shen Y.. (2020). CT quantification and machine-learning models for assessment of disease severity and prognosis of COVID-19 patients. Acad. Radiol. 27, 1665–1678. doi: 10.1016/j.acra.2020.09.004, PMID: 33046370 PMC7505599

[ref5] Camporota L. Cronin J. N. Busana M. Gattinoni L. Formenti F. (2022). Pathophysiology of coronavirus-19 disease acute lung injury. Curr. Opin. Crit. Care 28, 9–16. doi: 10.1097/MCC.0000000000000911, PMID: 34907979 PMC8711311

[ref6] Caso F. Costa L. Ruscitti P. Navarini L. Del Puente A. Giacomelli R.. (2020). Could Sars-coronavirus-2 trigger autoimmune and/or autoinflammatory mechanisms in genetically predisposed subjects? Autoimmun. Rev. 19:102524. doi: 10.1016/j.autrev.2020.102524, PMID: 32220633 PMC7271072

[ref7] Chen R. Sang L. Jiang M. Yang Z. Jia N. Fu W.. (2020). Longitudinal hematologic and immunologic variations associated with the progression of COVID-19 patients in China. J. Allergy Clin. Immunol. 146, 89–100. doi: 10.1016/j.jaci.2020.05.003, PMID: 32407836 PMC7212968

[ref8] Chen N. Zhou M. Dong X. Qu J. Gong F. Han Y.. (2020). Epidemiological and clinical characteristics of 99 cases of 2019 novel coronavirus pneumonia in Wuhan, China: a descriptive study. Lancet 395, 507–513. doi: 10.1016/S0140-6736(20)30211-7, PMID: 32007143 PMC7135076

[ref9] Chung H. Ko H. Kang W. S. Kim K. W. Lee H. Park C.. (2021). Prediction and feature importance analysis for severity of COVID-19 in South Korea using artificial intelligence: model development and validation. J. Med. Internet Res. 23:e27060. doi: 10.2196/27060, PMID: 33764883 PMC8057199

[ref10] Del Valle D. M. Kim-Schulze S. Huang H. H. Beckmann N. D. Nirenberg S. Wang B.. (2020). An inflammatory cytokine signature predicts COVID-19 severity and survival. Nat. Med. 26, 1636–1643. doi: 10.1038/s41591-020-1051-9, PMID: 32839624 PMC7869028

[ref11] Fatima N. Khokhar S. A. Farooq Ur Rehman R. M. (2023). Correlation between oxygen saturation of patient and severity index of Covid 19 pneumonia on CT. J. Pak. Med. Assoc. 73, 60–63. doi: 10.47391/JPMA.5586, PMID: 36842008

[ref12] Fu J. Kong J. Wang W. Wu M. Yao L. Wang Z.. (2020). The clinical implication of dynamic neutrophil to lymphocyte ratio and D-dimer in COVID-19: a retrospective study in Suzhou China. Thromb. Res. 192, 3–8. doi: 10.1016/j.thromres.2020.05.006, PMID: 32407937 PMC7201241

[ref13] Gallais F. Velay A. Nazon C. Wendling M. J. Partisani M. Sibilia J.. (2021). Intrafamilial exposure to SARS-CoV-2 associated with cellular immune response without seroconversion, France. Emerg. Infect. Dis. 27, 113–121. doi: 10.3201/eid2701.203611, PMID: 33261718 PMC7774579

[ref14] Huang C. Wang Y. Li X. Ren L. Zhao J. Hu Y.. (2020). Clinical features of patients infected with 2019 novel coronavirus in Wuhan, China. Lancet 395, 497–506. doi: 10.1016/S0140-6736(20)30183-5, PMID: 31986264 PMC7159299

[ref15] Liu J. Li S. Liu J. Liang B. Wang X. Wang H.. (2020). Longitudinal characteristics of lymphocyte responses and cytokine profiles in the peripheral blood of SARS-CoV-2 infected patients. EBioMedicine 55:102763. doi: 10.1016/j.ebiom.2020.102763, PMID: 32361250 PMC7165294

[ref16] Liu F. Zhang Q. Huang C. Shi C. Wang L. Shi N.. (2020). CT quantification of pneumonia lesions in early days predicts progression to severe illness in a cohort of COVID-19 patients. Theranostics 10, 5613–5622. doi: 10.7150/thno.45985, PMID: 32373235 PMC7196293

[ref17] Matsubara S. Sudo K. Kushimoto K. Yoshii R. Inoue K. Kinoshita M.. (2024). Prediction of acute lung injury assessed by chest computed tomography, oxygen saturation/fraction of inspired oxygen ratio, and serum lactate dehydrogenase in patients with COVID-19. J. Infect. Chemother. 30, 406–416. doi: 10.1016/j.jiac.2023.11.013, PMID: 37984540

[ref18] National Health Commission (2023). NHC of the PRC and National Administration of Traditional Chinese Medicine of the PRC. 10th version of the National Health Commission of China’s guidelines for diagnosis and treatment of novel coronavirus. Beijing: National Health Commission.

[ref19] Pang B. Li H. Liu Q. Wu P. Xia T. Zhang X.. (2021). CT quantification of COVID-19 pneumonia at admission can predict progression to critical illness: a retrospective multicenter cohort study. Front. Med. 8:689568. doi: 10.3389/fmed.2021.689568, PMID: 34222293 PMC8245676

[ref20] Poston J. T. Patel B. K. Davis A. M. (2020). Management of Critically ill Adults with COVID-19. JAMA 323, 1839–1841. doi: 10.1001/jama.2020.4914 32215647

[ref21] Pu J. Leader J. K. Bandos A. Ke S. Wang J. Shi J.. (2021). Automated quantification of COVID-19 severity and progression using chest CT images. Eur. Radiol. 31, 436–446. doi: 10.1007/s00330-020-07156-2, PMID: 32789756 PMC7755837

[ref22] Qadir N. Sahetya S. Munshi L. Summers C. Abrams D. Beitler J.. (2024). An update on Management of Adult Patients with acute respiratory distress syndrome: an official American Thoracic Society clinical practice guideline. Am. J. Respir. Crit. Care Med. 209, 24–36. doi: 10.1164/rccm.202311-2011ST, PMID: 38032683 PMC10870893

[ref23] Qin R. He L. Yang Z. Jia N. Chen R. Xie J.. (2023). Identification of parameters representative of immune dysfunction in patients with severe and fatal COVID-19 infection: a systematic review and Meta-analysis. Clin. Rev. Allergy Immunol. 64, 33–65. doi: 10.1007/s12016-021-08908-8, PMID: 35040086 PMC8763427

[ref24] Ranieri V. M. Rubenfeld G. D. Thompson B. T. Ferguson N. D. Caldwell E. Fan E.. (2012). Acute respiratory distress syndrome: the Berlin Definition. JAMA 307, 2526–2533. doi: 10.1001/jama.2012.5669 22797452

[ref25] Salahshour F. Mehrabinejad M. M. Nassiri Toosi M. Gity M. Ghanaati H. Shakiba M.. (2021). Clinical and chest CT features as a predictive tool for COVID-19 clinical progress: introducing a novel semi-quantitative scoring system. Eur. Radiol. 31, 5178–5188. doi: 10.1007/s00330-020-07623-w, PMID: 33449185 PMC7809225

[ref26] Santus P. Radovanovic D. Saderi L. Marino P. Cogliati C. De Filippis G.. (2020). Severity of respiratory failure at admission and in-hospital mortality in patients with COVID-19: a prospective observational multicentre study. BMJ Open 10:e043651. doi: 10.1136/bmjopen-2020-043651 PMC7549463 33040020

[ref27] Shaikh F. Andersen M. B. Sohail M. R. Mulero F. Awan O. Dupont-Roettger D.. (2021). Current landscape of imaging and the potential role for artificial intelligence in the management of COVID-19. Curr. Probl. Diagn. Radiol. 50, 430–435. doi: 10.1067/j.cpradiol.2020.06.009, PMID: 32703538 PMC7320858

[ref28] Sudre C. H. Murray B. Varsavsky T. Graham M. S. Penfold R. S. Bowyer R. C.. (2021). Attributes and predictors of long COVID. Nat. Med. 27, 626–631. doi: 10.1038/s41591-021-01292-y, PMID: 33692530 PMC7611399

[ref29] Tanaka H. Maetani T. Chubachi S. Tanabe N. Shiraishi Y. Asakura T.. (2023). Clinical utilization of artificial intelligence-based COVID-19 pneumonia quantification using chest computed tomography - a multicenter retrospective cohort study in Japan. Respir. Res. 24:241. doi: 10.1186/s12931-023-02530-2, PMID: 37798709 PMC10552312

[ref30] Tobin M. J. Laghi F. Jubran A. (2020). Why COVID-19 silent hypoxemia is baffling to physicians. Am. J. Respir. Crit. Care Med. 202, 356–360. doi: 10.1164/rccm.202006-2157CP, PMID: 32539537 PMC7397783

[ref31] Torres-Castro R. Vasconcello-Castillo L. Alsina-Restoy X. Solis-Navarro L. Burgos F. Puppo H.. (2021). Respiratory function in patients post-infection by COVID-19: a systematic review and meta-analysis. Pulmonology 27, 328–337. doi: 10.1016/j.pulmoe.2020.10.013, PMID: 33262076 PMC7687368

[ref32] Wang L. Liu T. Yue H. Zhang J. Sheng Q. Wu L.. (2023). Clinical characteristics and high risk factors of patients with omicron variant strain infection in Hebei, China. Front. Cell. Infect. Microbiol. 13:1294904. doi: 10.3389/fcimb.2023.1294904, PMID: 38145047 PMC10744887

[ref33] Wasilewski P. G. Mruk B. Mazur S. Półtorak-Szymczak G. Sklinda K. Walecki J. (2020). COVID-19 severity scoring systems in radiological imaging - a review. Pol. J. Radiol. 85, e361–e368. doi: 10.5114/pjr.2020.98009, PMID: 32817769 PMC7425223

[ref34] Xu L. Raitoharju J. Iosifidis A. Gabbouj M. (2022). Saliency-based multilabel linear discriminant analysis. IEEE Trans. Cybern. 52, 10200–10213. doi: 10.1109/TCYB.2021.3069338, PMID: 33877998

[ref35] Xu Z. Shi L. Wang Y. Zhang J. Huang L. Zhang C.. (2020). Pathological findings of COVID-19 associated with acute respiratory distress syndrome. Lancet Respir. Med. 8, 420–422. doi: 10.1016/S2213-2600(20)30076-X, PMID: 32085846 PMC7164771

[ref36] Zhang L. Chen H. Zhang L. Chen X. (2021). Analysis of the risk factors for progression from mild to severe cases of COVID-19. Prev. Med. 4, 751–764.

[ref37] Zhang K. Liu X. Shen J. Li Z. Sang Y. Wu X.. (2020). Clinically applicable AI system for accurate diagnosis, quantitative measurements, and prognosis of COVID-19 pneumonia using computed tomography. Cell 181, 1423–1433.e11. doi: 10.1016/j.cell.2020.04.045, PMID: 32416069 PMC7196900

[ref38] Zhao Q. Meng M. Kumar R. Wu Y. Huang J. Deng Y.. (2020). Lymphopenia is associated with severe coronavirus disease 2019 (COVID-19) infections: a systemic review and meta-analysis. Int. J. Infect. Dis. 96, 131–135. doi: 10.1016/j.ijid.2020.04.086, PMID: 32376308 PMC7196544

[ref39] Zhou W. Liu Y. Xu B. Wang S. Li S. Liu H.. (2021). Early identification of patients with severe COVID-19 at increased risk of in-hospital death: a multicenter case-control study in Wuhan. J. Thorac. Dis. 13, 1380–1395. doi: 10.21037/jtd-20-2568, PMID: 33841931 PMC8024856

